# Genetic Code Expansion of *Vibrio natriegens*

**DOI:** 10.3389/fbioe.2021.594429

**Published:** 2021-02-26

**Authors:** Eden Ozer, Lital Alfonta

**Affiliations:** Department of Life Sciences, Department of Chemistry and Ilse Katz Institute for Nanoscale Science and Technology, Ben-Gurion University of the Negev, Beer-Sheva, Israel

**Keywords:** *Vibrio natriegens*, genetic code expansion, unnatural amino acids, propargyl-*L*-lysine, synthetic biology

## Abstract

*Escherichia coli* has been considered as the most used model bacteria in the majority of studies for several decades. However, a new, faster chassis for synthetic biology is emerging in the form of the fast-growing gram-negative bacterium *Vibrio natriegens*. Different methodologies, well established in *E. coli*, are currently being adapted for *V. natriegens* in the hope to enable a much faster platform for general molecular biology studies. Amongst the vast technologies available for *E. coli*, genetic code expansion, the incorporation of unnatural amino acids into proteins, serves as a robust tool for protein engineering and biorthogonal modifications. Here we designed and adapted the genetic code expansion methodology for *V. natriegens* and demonstrate an unnatural amino acid incorporation into a protein for the first time in this organism.

## Introduction

*Vibrio natriegens* (*V. natriegens*), is a fast-growing gram-negative bacterium identified in the 1960s of the 20th century with a remarkable division time of less than 10 min (Eagon, 1962). Despite the fact that *Escherichia coli* (*E. coli*) is considered the most convenient organism for lab work, it has a division time that is roughly double that of *V. natriegens* (Clark and Maaløe, 1967). Considering the need to optimize the efficiency of molecular and synthetic biology studies in the lab, *V. natriegens* presents an unprecedented opportunity to speed-up lab work and emerges as a novel synthetic biology and biotechnological chassis. Apart from its rapid growth, *V. natriegens* can also reach higher cell densities than *E. coli*, it can tolerate a broad range of pH conditions, possess natural competence for environmental DNA receival, utilize a wide range of substrate and grow on various carbon sources, has a higher substrate uptake, can serve as a safer expression host for pathogen related proteins and express proteins in high yields. Recently, multiple studies have emerged, demonstrating the adaptation of methodologies used in *E. coli*, for their utilization in *V. natriegens* (Hoff et al., 2020; Peng et al., 2020). As such, protein expression (Weinstock et al., 2016; Schleicher et al., 2018), molecular biology (Weinstock et al., 2016; Dalia et al., 2017; Tschirhart et al., 2019), and cell-free protein expression platforms (Des Soye et al., 2018; Failmezger et al., 2018; Wiegand et al., 2018) were successfully developed in *V. natriegens*.

The incorporation of unnatural amino acids (Uaas) into proteins is part of the genetic code expansion experimental efforts and is considered a powerful tool in protein engineering (Xiao and Schultz, 2016). The insertion of a Uaa in a site-specific manner allows the introduction of new functions in a protein in a controlled manner and their use in research that involves fluorescence resonance energy transfer (FRET) (Ozer et al., 2017), transcription/translation kinetic analysis (Schlesinger et al., 2017), engineering of oxygen tolerant enzymes (Bhagat et al., 2020), and cellular localization (Cohen et al., 2020). To date, over 200 Uaas were incorporated into different proteins (Xiao and Schultz, 2016) in different organisms including the primary *E. coli* system, amongst them are Salmonella (Gan et al., 2016), cyanobacteria (Chemla et al., 2017), Yeast (Hancock et al., 2010), *Caenorhabditis elegans* (Greiss and Chin, 2011), mouse (Ernst et al., 2016), mammalian cells (Italia et al., 2017), and cell-free systems (Ozer et al., 2017). While evolving into a common biotechnological platform, it is yet to be adapted to *V. natriegens*. Genetic code expansion in *V. natriegens* can serve as an improved and a faster platform for future protein engineering in this new chassis, possibly for much-needed research in the organism itself but mainly as a powerhouse for production of proteins with expanded amino acid repertoire.

## Materials and Methods

### Reagents

Propargyl-L-lysine (PrK) was purchased from Syn- Chem (Elk Grove Village, IL, United States). Tris(3-hydroxypropyltriazolylmethyl) amine (THPTA), Tetramethyl- rhodamine-azide (TAMRA-Az), and TAMRA-alkyne were purchased from Sigma-Aldrich (Rehovot, Israel). All restriction enzymes were purchased from Thermo Fisher Scientific (Waltham, MA, United States), while all DNA oligonucleotides were obtained from Syntezza Bioscience (Jerusalem, Israel).

### Bacterial Strains

All *E. coli* molecular biology experiments in this study were performed in DH5a strain while all *V. natriegens* molecular biology was performed in ATCC strain # 14048.

### Plasmids Construction

pBEST-P70b-UTR1-deGFP-6xHis-T500 was used as the basis for all plasmid designs (Schlesinger et al., 2017). All PCR generated amplicons were separated on an agarose gel and purified using nucleospin gel and PCR clean-up (Macherey-Nagel, Germany).

The final plasmid contains the *Methanosarcina mazei* pyrrolysine system (*Mm*Pyl), composed of *Mm*Pyl-tRNA and *Mm*Pyl tRNA-synthetase (*Mm*PylRS), and was constructed in a two-step procedure. The first plasmid that was generated was with the addition of *Mm*Pyl-tRNA to the pBEST vector, and was named pRaGE-Pyl tRNA plasmid. tRNA native promoter and terminator regions were chosen from the *V. natriegens* genome and were amplified using primers 1 + 2 and 3 + 4 (Supplementary Table 2). *Mm*Pyl-tRNA was generated through overlapping segments between primers 2 and 3 using a Gibson assembly. pBEST vector amplification was performed using primers 5 + 6 (Supplementary Table 2) and *Dpn*I restriction enzyme. Following a Gibson assembly, the assembly mix was transformed into DH5a *E. coli* cells using a standard heat-shock protocol and plated on selective media (100 μg/mL carbenicillin).

The next plasmid generation was pRaGE harboring the full *Mm*Pyl system named pRaGE-Pyl: *Mm*Pyl-tRNA and *Mm*PylRS together. The design was as an extension to the previously generated pRaGE-Pyl tRNA variant. Vector amplicon was generated using primers 6 + 7 (Supplementary Table 2) and underwent *Dpn*I treatment. Yeast origin of replication and selection marker (designed for yeast assembly) were amplified using primers 8 + 9 (Supplementary Table 2). *V. natriegens* native regions upstream and downstream of the glutamic acid tRNA synthetase (GluRS) were chosen to surround the *Mm*PylRS gene. GluRS promoter and terminator were amplified from *V. natriegens* genome using primer 10 + 11 and 12 + 13, respectively (Supplementary Table 2). Finally, the *Mm*PylRS fragment was amplified using primers 14 + 15 (Supplementary Table 2). Standard yeast assembly protocol (Chemla et al., 2017) was performed for all amplicons. The assembled plasmid was extracted from yeast (ZYMO research, Irvine, CA, United States) and was transformed into DH5a *E. coli* using a standard heat-shock protocol and plated on selective media (100 μg/mL carbenicillin).

The final plasmid assembly was performed in order to generate a 2nd pRaGE plasmid harboring the partial OTS, this time with *Mm*PylRS only, named pRaGE-PylRS. Using the previously generated pRaGE-Pyl plasmid, containing the full OTS, the fragment was amplified using primers 5 + 16 (Supplementary Table 2), assembled by a yeast assembly. All plasmids were sequenced for proper assembly validation.

### Electrocompetent Cells Preparation and Electroporation

Electroporation protocol was based on a published protocol by Lee et al. (2019). Liquid culture was grown in LB3 (LB-Miller with extra salt for final NaCl concentration of 3%) at 30°C for overnight incubation. For each transformation, 100 μL of overnight culture were pelleted down and resuspended with 100 μL of fresh LB3 and added to 10 mL of LB3. Subculture was grown at 37°C for 1:10 h (approximately at a 0.4 OD_600_) and harvested at 4°C, 3,500 × *g* for 5 min. From this point on, all steps were performed on ice. Cells were resuspended with 1 M cold sorbitol and transferred into a chilled 1.5 mL Eppendorf tube where they were pelleted down at 4°C, 20,000 × *g* for 1 min. Cells were then washed twice more with 1 M cold sorbitol and 4°C, 20,000 × *g* for 1 min centrifugation. Finally, electrocompetent cells were resuspended with 50 μL 1 M cold sorbitol and used fresh for electroporation.

Approximately 100 ng of the desired plasmid were added to the electrocompetent cells and stirred gently. Following 2 min incubation on ice, cells were then transferred into a chilled 1 mm cuvette (Cell Projects Ltd., Kent, United Kingdom) and left on ice for five additional minutes. Cells were electroporated using Gene Pulser^®^ II (Bio-Rad, Hercules, CA, United States) using the following parameters: 0.4 kV, 1 kΩ, 25 μF and were immediately recovered with 1 mL LB3 media for 1–2 h at 37°C. Cells were plated on selective LB3 agar plates carrying 100 μg/mL carbenicillin. Plates were grown at 30°C overnight.

### Protein Expression and Cell Lysis

A swab from a glycerol stock was grown in LB3 at 30°C overnight (when bacteria were harboring a plasmid, carbenicillin was added to a final concentration of 100 μL/mL). Fresh culture of 3 mL LB3 was made from previously grown stationery culture using 1:100 dilution and incubated at 30°C overnight. Cultures were supplemented with a final concentration of 1 mM PrK. Following liquid culture growth, 1 mL of the culture was lysed. The culture was then washed once with 1 mL of phosphate buffer 100 mM pH = 7. The cells were pelleted down and resuspended with 100 μL lysis solution composed of 90% phosphate buffer, 10% BugBuster^®^ 10X protein extraction reagent (Merck, Billerica, MA, United States), Turbonuclease (Sigma, St. Louis, MO, United States), Lysozyme (Sigma-Aldrich, Rehovot, Israel) and protease inhibitor (Merck, Darmstadt, Germany). Samples were incubated at 25°C for 30 min with mild shaking, followed by 4°C centrifugation at 10,000 × *g* for 10 min. Supernatant was collected for further analysis.

### SDS-PAGE and Western-Blot Analyses

Lysed samples were separated using SDS-PAGE 4–20% Expressplus protein gel (GeneScript, Nanjing, China). Transferring gel stamp to an Immun-Blot^®^ PVDF membrane (Bio-Rad, Hercules, CA, United States) using eBlot^®^ protein transfer system (GenScript, Nanjing, China), samples underwent a standard Western-blot protocol with GFP (B-2) mouse monoclonal antibody (sc-9996, Santa Cruz, CA, United States) as primary antibody and Goat anti-mouse IgG H&L (HRP) (ab97023, abcam, Cambridge, United Kingdom) as a secondary antibody. Membrane imaging was done using ImageQuant LAS 4000 imager with a maximum exposure time of 10 s (Fujifilm, Tokyo, Japan).

### Suppression Efficiency Calculation

**FIGURE 1:**
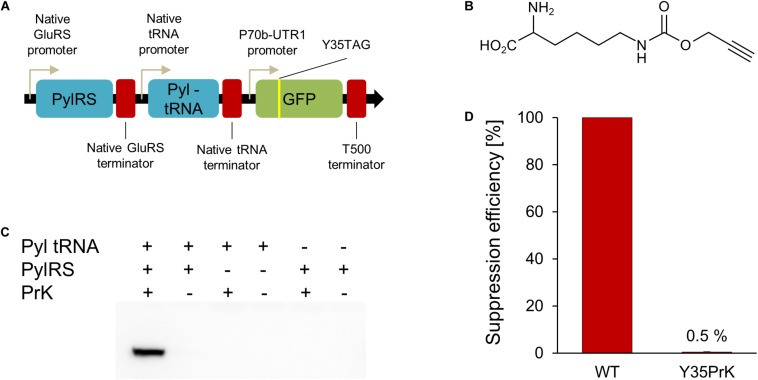
*V. natriegens* genetic code expansion system. **(A)** Genetic map of constructed pRaGE-Pyl. **(B)** Unnatural amino acid (Uaa) used in this study, propargyl-L-lysine (PrK). **(C)** Anti-GFP Western-blot analysis to test orthogonality and Uaa incorporation into GFP. **(D)** Calculated suppression efficiency for genetic code expansion in *V. natriegens* based on GFP Y35PrK mutant compared to WT GFP.

Three separate sets of liquid cultures were grown at 30°C for 16 h. Each set was composed of three *V. natriegens* strains: native strain, wild type (WT) GFP expression strain and TAG mutant GFP expression strain grown in the presence of 1 mM PrK. All samples were tested for OD_600_ and fluorescence (488 nm excitation and 507 nm emission) in three technical repeats, using Synergy HT plate reader (Biotek, Winooski, VT, United States). Each sample fluorescence value was divided by its OD_600_ value. Values of WT and mutant expression were normalized to the native strain’s value of fluorescence/OD_600_. Finally, the suppression efficiency of each set out of the three was determined through the value of normalized fluorescence/OD_600_ of mutant divided by the value of normalized fluorescence/OD_600_ of WT. Error bar in Figure 1D could only be calculated for mutant expression (since WT value was always set to 100% by definition), representing a standard deviation between three different suppression efficiencies values calculated to be ± 0.037%.

### Protein Purification, Coomassie Staining, and Peptide Mass Fingerprinting

A 100 mL of LB3 culture of *V. natriegens* harboring the pRaGE-Pyl TAG-deGFP-Y35TAG-NHis plasmid, was grown at 30°C overnight. The culture was lysed using standard needle sonication, followed by IMAC purification (Novagene, Madison, WI, United States) according to manufacturer guidelines. The elution fraction was concentrated using Vivaspin 6, 10000 MWCO PES (Sartorius, Goettingen, Germany). Purification products were run on SDS-PAGE 4–20% Expressplus protein gel (GeneScript, Nanjing, China) and a standard Coomassie staining protocol.

A band of ∼27 kDa corresponding to GFP size was incised and digested in-gel by trypsin according to manufacturer’s protocol (Promega). Peptides were then extracted from the gel and loaded onto a liquid chromatography mass spectrometer (LC-MS). LC-MS analysis was performed using an Eksigent nano−HPLC (model nanoLC-2D, Netherlands) connected to an LTQ Orbitrap XL ETD (Thermo Fisher Scientific, Germany and United States). Reverse−phase chromatography of peptides was performed using a C−18 column [IntegraFrit^TM^, 360 μm OD × 75 ID (μm); New Objective United States]. Peptides were separated by a 70 min linear gradient, starting with 100% buffer A (5% acetonitrile, 0.1% formic acid) and ending with 80% buffer B (80% acetonitrile, 0.1% formic acid), at a flow rate of 300 mL/min. A full scan, acquired at 60,000 resolution, was followed by CID MS/MS analysis performed for the five most abundant peaks, in a data−dependent mode. Fragmentation (with minimum signal trigger threshold set at 500) and detection of fragments were carried out in a linear ion trap. Maximum ion fill time settings were 500 ms for a high−resolution full scan in an Orbitrap analyzer and 200 ms for MS/MS analysis in the ion trap. The AGC settings were 5 × 105 and 1 × 104 (MS/MS) for Orbitrap and linear ion trap analyzers, respectively. Proteins were identified and validated using SEQUEST software and data sets operated under the Proteome Discoverer 2.3 software (Thermo Fisher Scientific). Mass tolerance for precursors and fragmentations was set to 10 ppm and 0.8 Da, respectively.

### Click Reaction and Fluorescent Imaging

Cu(I)-catalyzed azide-alkyne cycloaddition (CuAAC) click reaction (Kolb et al., 2001) was performed on purified GFP Y35PrK protein for PrK incorporation validation. Fluorescent dye was added to a concentration of 50 μM, while THPTA, sodium ascorbate, and CuCl_2_ were added to a final concentration of 1.2 mM, 2.5 mM, and 200 μM, respectively. A volume of 38 μL concentrated purified protein was added to the reaction, followed by 1 h incubation at room temperature with mild shaking. After the reaction, samples were examined through SDS-PAGE 4–20% Expressplus protein gel (GeneScript, Nanjing, China). Fluorescent SDS-PAGE images were obtained using Typhoon FLA 9500 imager (GE Healthcare Life Sciences, Uppsala, Sweden) 532 nm laser and LPG green filter on TAMRA setting. Following fluorescence imaging, the experiment has proceeded to anti-GFP Western-blot analysis as was described earlier.

## Results and Discussion

In order to establish Uaa incorporation in proteins in an organismfor the first time, an orthogonal translation system (OTS) composed of a tRNA and tRNA-synthetase pair is needed (Xiao and Schultz, 2016). In order to confirm the orthogonality of the OTS in *V. natriegens*, the OTS needs to interact with a specific Uaa and to have no cross-reactivity with other components: native amino acids, endogenous tRNAs or tRNA-synthetases. The *Methanosarcina mazei* pyrrolysyl (*Mm*Pyl) OTS was employed and proven orthogonal in several gram-negative bacteria (Greiss and Chin, 2011; Chemla et al., 2017; Ozer et al., 2017). In addition, the *Mm*Pyl OTS was demonstrated to incorporate a large variety of Uaas and was therefore chosen to be tested in *V. natriegens* as well (Dumas et al., 2015). In order for the OTS to be expressed under the regulation of native promoters and terminators, the upstream and downstream sequences of genomic regions of *V. natriegens* were used. According to the genomic analysis on codon usage in *V. natriegens* (Lee et al., 2019), we have found that the GAA codon, encoding for glutamic acid, is the most abundant codon in the genome of this organism. Since promoters and terminators are not yet fully annotated and characterized in *V. natriegens*, we have used an educated guess as of their identity. Hence, glutamic acid tRNA-synthetase (GluRS) upstream and downstream regions were cloned before and after *Mm*Pyl tRNA-synthetase (*Mm*PylRS) gene, respectively. In addition, the upstream and downstream regions of a sequence of three native *V. natriegens*’s tRNAs (glutamyl-tRNA, lysyl-tRNA, and valyl-tRNA) were chosen to control the expression of *Mm*Pyl-tRNA.

The *Mm*Pyl-tRNA anti-codon loop was to correspond to a TAG stop-codon mutation encoded in a gene of interest, specifically, a TAG mutation was introduced to a deGFP reporter gene. The TAG mutation site was introduced instead of a tyrosine at position 35 of GFP, a known permissive site (Chemla et al., 2018) for proteins in *E. coli*. Based on reports that *V. natriegens* can harbor plasmids with the pBR322 origin of replication (Tschirhart et al., 2019), the pBEST plasmid (Schlesinger et al., 2017) was used as a vector encoding for the *Mm*Pyl OTS and mutant GFP gene, which were combined into a new plasmid which name reflects *V. natriegens* fast-growing nature: rapid genetic code expansion plasmid (pRaGE-Pyl, Figure 1A) (the full plasmid sequence is available in the Supplementary Material section).

The Uaa that we have chosen to incorporate into GFP was propargyl-L-lysine (PrK, Figure 1B). We have first tested the effect of PrK on *V. natriegens* growth. PrK supplement as well as pRaGE variants transformation into *V. natriegens* did not impair bacterial growth (Supplementary Figures 1, 2, respectively), hence orthogonality and incorporation validation were followed. The best way to test orthogonality is by testing each OTS component separately. For that purpose, two additional variants of the pRaGE-Pyl plasmid were generated and named pRaGE-Pyl tRNA and pRaGE-PylRS. Both variants encode for the mutant GFP reporter protein, as in the original pRaGE-Pyl construct, however, each variant carried only a single component out of the orthogonal translational pair, either orthogonal *Mm*Pyl-tRNA or orthogonal *Mm*PylRS. Protein expression was compared for all plasmid variants, either harboring the full system or a partial system, in the presence or absence of a Uaa.

Through its alkyne side group, PrK can be conjugated to an azide using a click reaction (Kolb et al., 2001). Ideally, we would have liked to perform click chemistry on *V. natriegens* whole cell lysate and determine site-specific PrK incorporation, along with orthogonality validation. This means that PrK should only be incorporated into GFP and not anywhere else in the proteome of the host organism. Unfortunately, click chemistry did not work on *V. natriegens* whole cell lysate, even when a purified protein, containing PrK, was added exogenously as a control. We speculate that an unknown component in the *V. natriegens* lysate is inhibiting the click reaction, and further research is needed to better understand the mechanism involved. Despite the fact that we could not confirm whether PrK is incorporated elsewhere in the proteome, it was still possible to determine the fidelity of PrK incorporation into GFP or whether it is one of the natural amino acids that was being incorporated instead. Upon GFP expression attempts, we have conducted a Western-blot analysis using an anti-GFP antibody. This analysis has established that only in the presence of the full *Mm*Pyl OTS and PrK, was a full-length GFP expressed (Figure 1C) (A complete Western-blot is available as Supplementary Figure 3). This result indicate that *Mm*Pyl OTS is orthogonal to the translational machinery of the host organism, as there was no full-length GFP expression when partial OTS was used or in the absence of a Uaa. Moreover, fully-elongated GFP expression was detected only in the presence of the entire OTS and PrK, suggesting positive Uaa incorporation as well and no misincorporation events.

In genetic code expansion, mutant protein yield is usually lower when compared to the wild type form (WT) and is commonly referred to as suppression efficiency. It is measured as the relative fraction of mutant protein levels, when incorporating Uaas, from WT protein production levels. Taking into consideration the context effects (Chemla et al., 2018), it is important to note that Uaa incorporation “events” are different from one another by multiple variables. For example, the context of the site of Uaa incorporation, target protein, codon usage, expression promoters and host organism may result in significant differences in protein yields. While protein quantification calculations are not very accurate and are affected by many different factors, for the time being *V. natriegens* genetic code expansion of GFP at the 35^th^ site, suppression efficiency was calculated to be ∼0.5% using GFP fluorescence (Figure 1D). Densitometry analysis of a Western blot with anti-GFP antibody of WT GFP and Y35TAG GFP mutant in the presence of PrK, although less accurate, resulted in a similar suppression efficiency as that determined by fluorescence measurements (Supplementary Figure 4). It is known that Uaa incorporation is a slower process than native amino acids incorporation, therefore mutant proteins are usually produced in lower quantities than WT proteins (Schlesinger et al., 2017). Also taking into consideration the use of endogenous promoters and terminators in this study, along with the fact that the *Mm*PylRS and GFP genes are not codon-optimized to the *V. natriegens* codon usage, could explain the observed low suppression efficiency. Presented here is the first attempt at genetic code expansion in *V. natriegens*, hence we believe the system can be further modified and optimized for future research. In the meantime, *V. natriegens* WT protein production is vast (Weinstock et al., 2016; Tschirhart et al., 2019), therefore the very low 0.5% suppression efficiency still resulted in the production of 104 μg of mutant protein from 100 mL of culture [calculations of protein yields were based on Ozer et al. (2017)]. Produced mutant protein was sufficient for downstream characterization and analyses.

**FIGURE 2:**
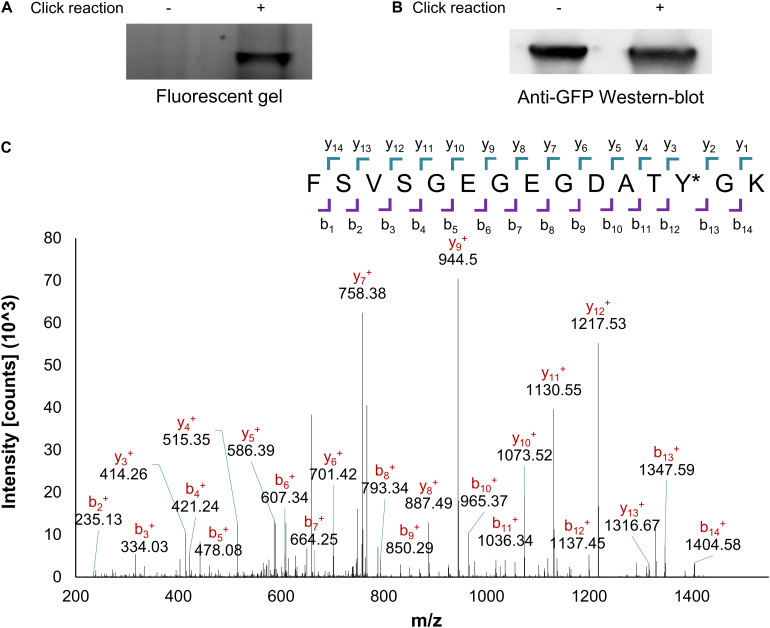
PrK incorporation validation. Purified protein with an incorporated alkyne bearing Uaa (GFP Y35PrK) with or without click reaction to an azide-bearing fluorophore analyzed through Fluorescent SDS-PAGE **(A)** or anti-GFP Western-blot analysis **(B)**. **(C)** Peptide mass fingerprinting fragmentation analysis of the peptide with incorporated PrK in the GFP protein, confirms the presence of PrK at the 35th position instead of tyrosine (labeled as Y^∗^, raw data available in Supplementary Material).

Mutant GFP protein, expressed in the presence of the complete OTS set and PrK, was purified and was further examined for validation of PrK incorporation. As mentioned, if incorporated, the alkyne moiety of PrK could undergo a click reaction. Using the click reaction to a TAMRA-azide fluorescent marker, a fluorescent SDS-PAGE band corresponded to PrK presence in the purified protein, a clear indication for Uaa incorporation (Figure 2A). Furthermore, the fluorescent band was ruled out as a possible artifact from the protein itself as there was no signal without a click reaction in the TAMRA-Az wavelengths, for the same protein concentrations in both samples identified by Western-blot (Figure 2B). Final validation for PrK incorporation into the purified mutant GFP was through peptide mass fingerprinting. The relevant peptide that included the 35th position of the protein with an incorporated Uaa, was identified with the correct modification of PrK instead of tyrosine in the WT proteins using MS/MS analysis (Figure 2C). These results, the azide conjugation and the peptide mass fingerprinting validated the desired incorporation of PrK in a site-specific manner and were therefore a direct proof for the desired genetic code expansion.

The results presented herein of PrK incorporation validation proved our ability to successfully expand the genetic code of *V. natriegens* for the first time. The system’s establishment was performed using a GFP reporter protein, yet now that the OTS proved functional it would be possible to employ it for other incorporation sites and target proteins in future research utilizing *V. natriegens*. As the employed system is the first one for genetic code expansion in *V. natriegens* it is expected to serve as a stepping stone for further developments and system optimization. However, in the meantime, despite a low calculated suppression efficiency, the amount of produced protein containing Uaa is satisfactory for protein purification and downstream analyses. Proteins with Uaas expressed in *V. natriegens* may replace production from *E. coli* and serve as a future tool for various research such as the production of electrochemically active enzymes, oxygen-tolerant enzymes, etc. As *V. natriegens* is being referred to as the most promising future chassis in synthetic biology, it holds great potential for promoting research in microbiology as well as convenient molecular biology, protein expression and biotechnological platform. The system presented herein has the potential to serve as a powerful tool and may be used for future, much-needed Uaa containing protein expression. We expect that genetic code expansion in *V. natriegens* will join previously developed methodologies in the growing tool-box relevant to this organism for the benefit of future research.

## Data Availability Statement

All additional data including primers and gene sequences, plasmid maps and additional validation results could be found in the supplementary material section. pRAGE plasmid is available through addgene with the following accession number and link: (https://www.addgene.org/160041/).

## Author Contributions

EO conceived, performed, analyzed experiments, and authored the manuscript. LA conceived experiments, supervised the research, provided facilities, written, and edited the manuscript. Both authors contributed to the article and approved the submitted version.

## Conflict of Interest

The authors declare that the research was conducted in the absence of any commercial or financial relationships that could be construed as a potential conflict of interest.
