# Supplementary material for: Genetic Code Expansion of Vibrio natriegens
Source: Front Bioeng Biotechnol. 2021 Feb 26;9:594429. doi: 10.3389/fbioe.2021.594429 (PMC7953155; doi:10.3389/fbioe.2021.594429)
Supplement: Supplementary file 1 [file Image_1.PDF]

Supplementary Material

Genetic Code Expansion of *Vibrio natriegens*

Eden Ozer, Lital Alfonta*

Departments of Life Sciences, Chemistry and Ilse Katz Institute for Nanoscale Science and Technology, Ben-Gurion University of the Negev, POBox 653, Beer-Sheva 8410501, Israel

*** Correspondence:**

Lital Alfonta

alfontal@bgu.ac.il

ORCID: ORCID: https://orcid.org/0000-0002-3805-8625

Table of Content:

Cover page		S-1
Table of content		S-2
Supplementary Figure 1	Viability in the presence of unnatural amino acid	S-3
Supplementary Figure 2	Viability with transformed plasmid	S-4
Supplementary Figure 3	Anti-GFP full Western-blot image	S-5
Supplementary Figure 4	Densitometry comparison of WT and mutant	S-6
Supplementary Figure 5	Peptide mass fingerprinting protein coverage	S-7
Supplementary Figure 6	pRaGE plasmid map	S-8
Supplementary Table 1	peptide mass fingerprinting peak list	S-9
Supplementary Table 2	Primers list	S-29
Gene sequences		S-32

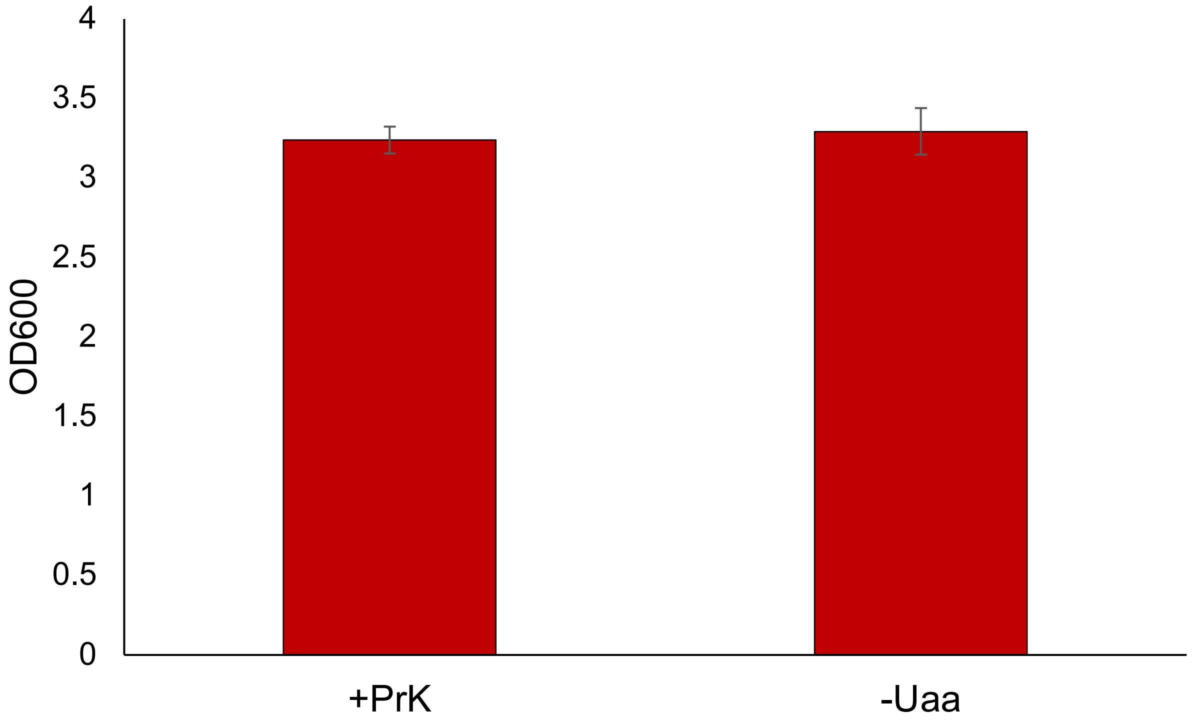

Supplementary Figure 1. Viability in the presence of unnatural amino acid used in this research. Native *V. natriegens* strain OD measurement after 16 hrs of growth in the presence or absence of unnatural amino acid (Uaa). Used Uaa was propargyl-L-lysine (PrK). Biomass growth is similar in both cultures.

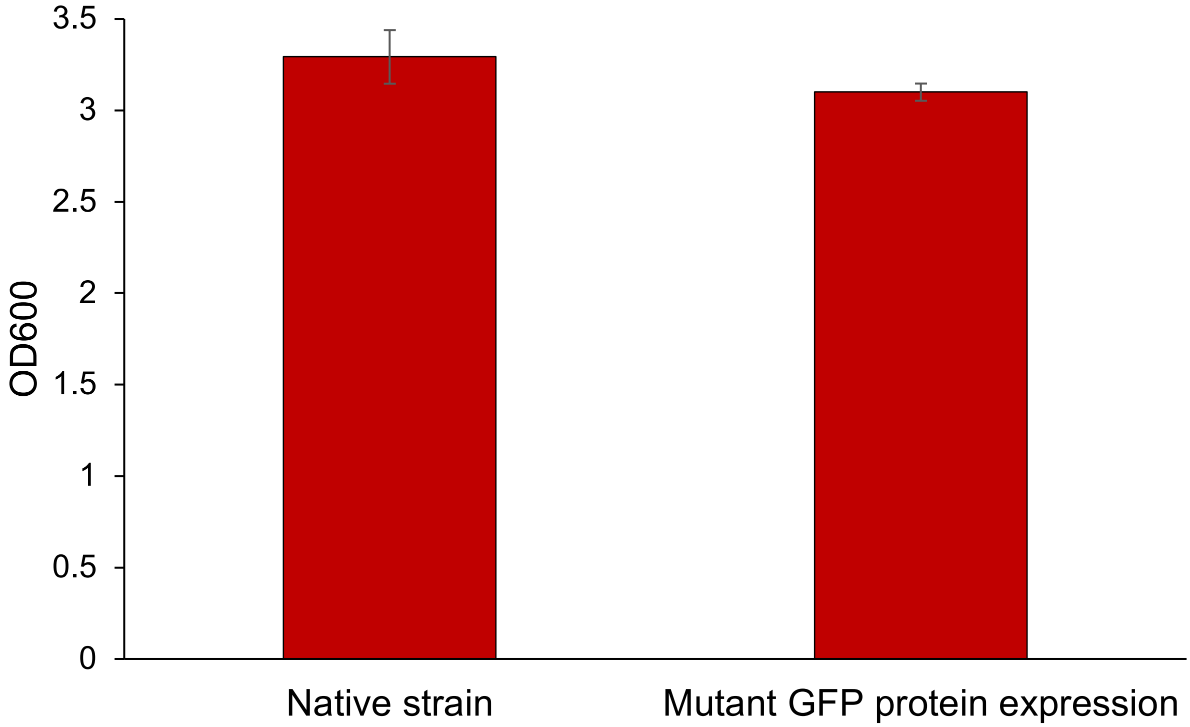

Supplementary Figure 2. Viability of native strain vs. plasmid bearing strain. OD measurement after 16 hrs of growth comparing between native strain and plasmid harboring strain for Uaa incorporated mutant GFP expression. Mutant growth and expression were performed in the presence of PrK and in the presence of 100 µg/mL carbenicillin.

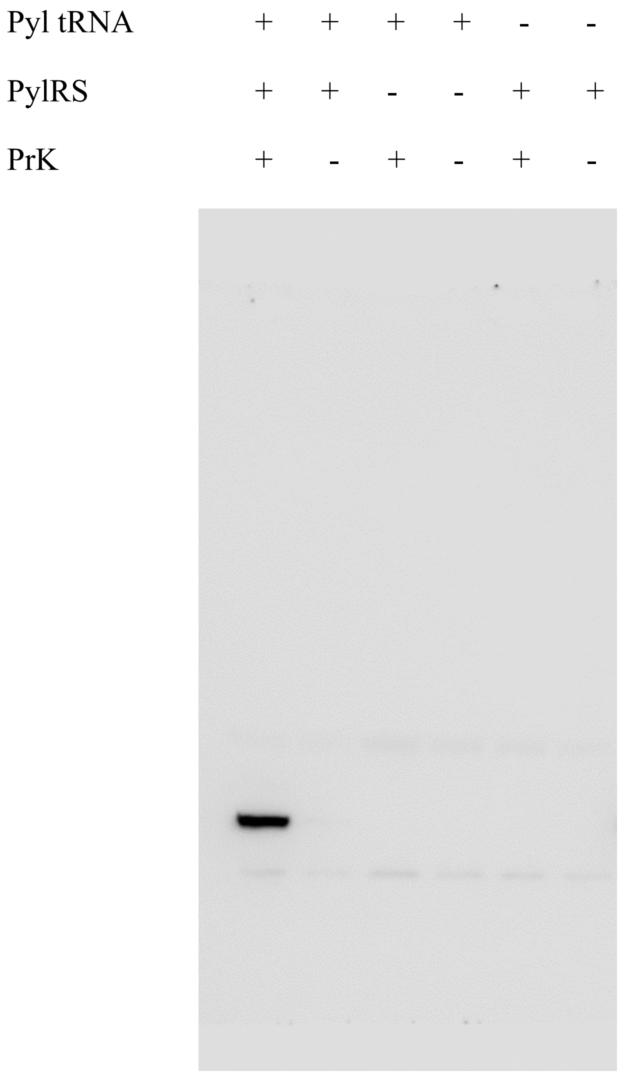

Supplementary Figure 3. Full image of Anti-GFP Western-blot analysis of orthogonality and Uaa incorporation into GFP from *V. natriegens*.

A

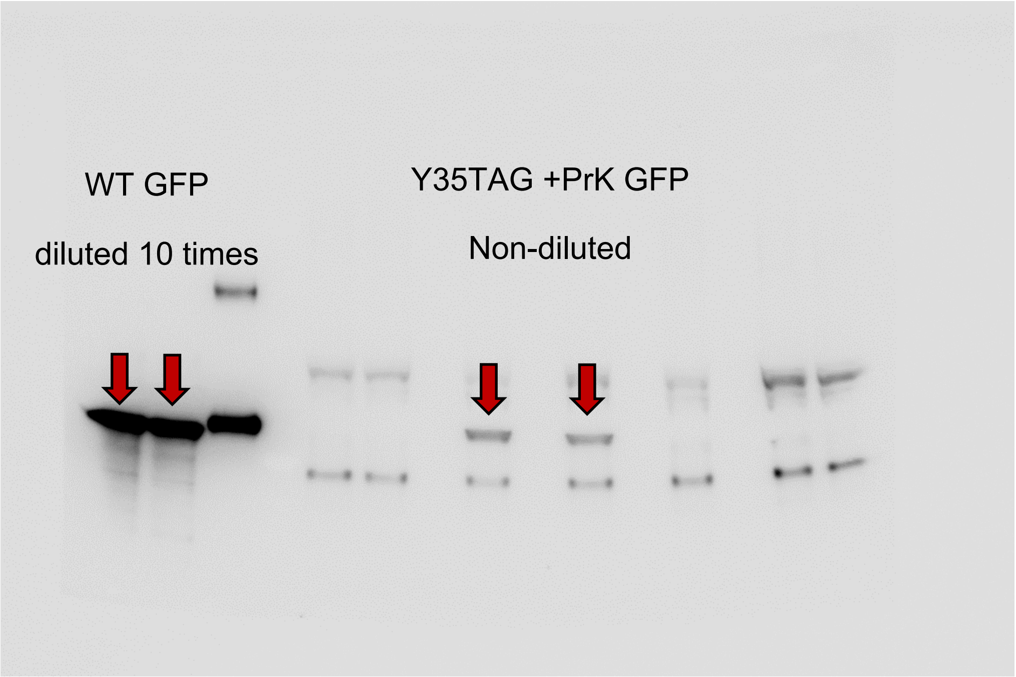

B

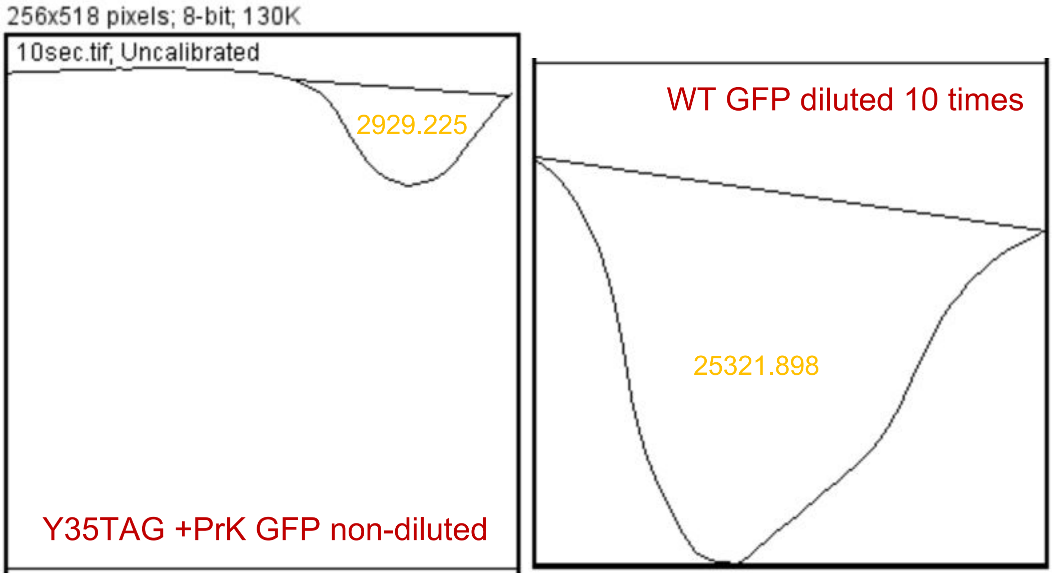

Supplementary Figure 4. (A) Full image of exemplary Anti-GFP Western-blot for comparison between WT GFP expression and Y35TAG mutant expression in the presence of PrK. WT protein sample was diluted 10 times prior to gel loading, while mutant protein was not diluted. (B) Exemplary densitometry analysis on WT GFP and Y35TAG mutant expression in the presence of PrK. Graphs correspond to 10 times diluted WT protein and non-diluted Y35TAG mutant protein in the presence of PrK.

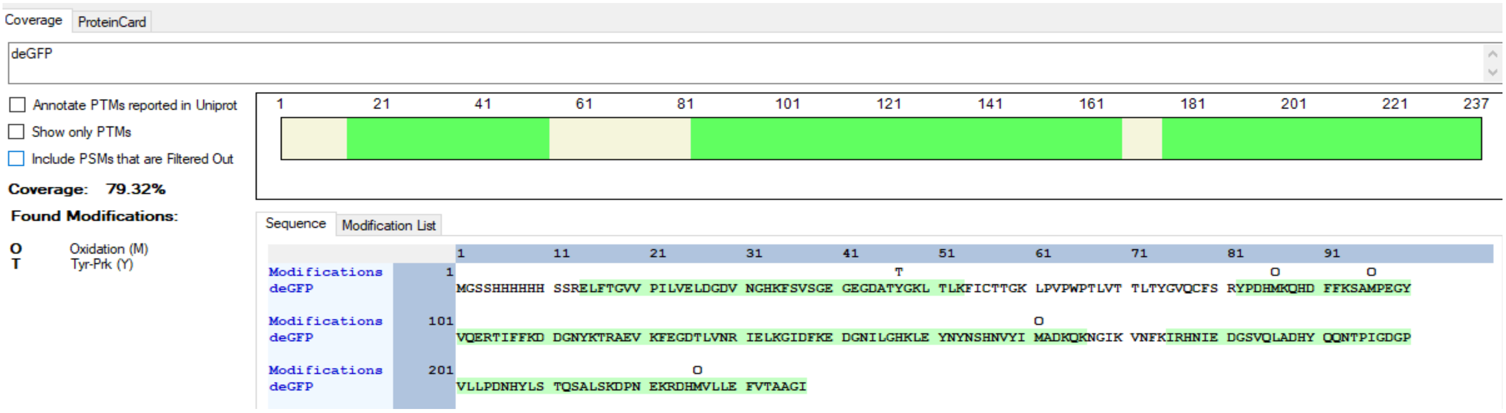

Supplementary Figure 5. Peptide mass fingerprinting protein coverage. Peptide mass fingerprinting coverage of identified peptides compared to the analyzed GFP protein.

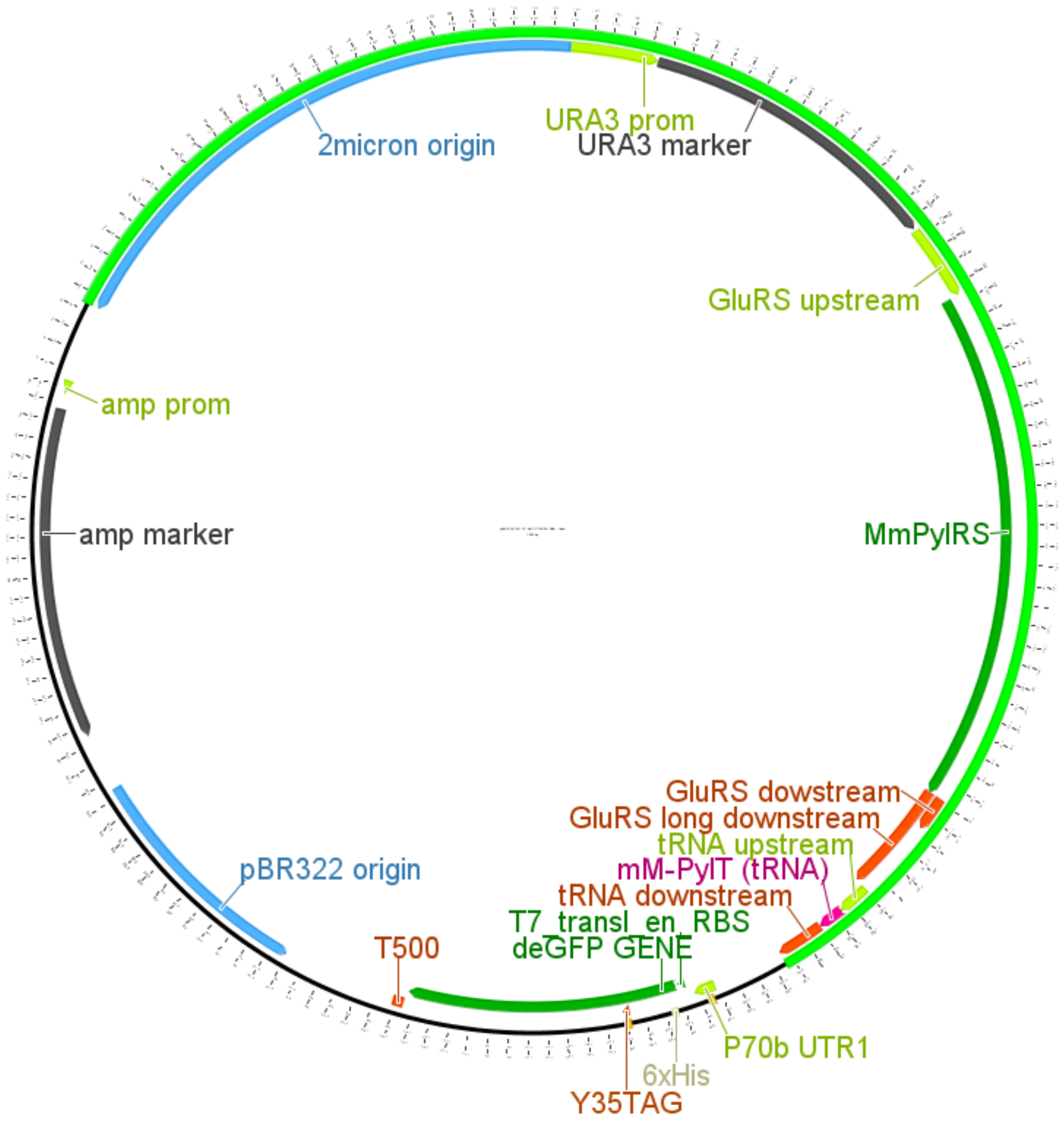

Supplementary Figure 6. pRaGE plasmid map. pRaGE construction illustration.

Supplementary Table 1. Peptide mass fingerprinting peak list

MK-4478-Lital-A-Eden--Gfp-Y35P-N13.RAW #925 RT: 19.4279 min			
ITMS, 775.8517@cid35.00, z=+2, Mono m/z=775.85187 Da, MH+=1550.69646 Da, Match Tol.=0.6 Da			
m/z	Intensity [counts]		
216.9635	176		
221.14178	220		
225.18057	94		
227.14514	117		
228.71927	80		
231.07495	55		
235.12627	1251	b_2^+	235.13
236.15157	29		
238.34503	496		
239.1048	1577	b_5^{2+}	239.1
240.08897	137		
241.16333	110		
242.1669	76		
243.26987	175		
243.93456	258		
245.18161	109		
246.01047	25		
247.07089	35		
249.46353	22		
250.14352	780	y_4^{2+}-NH_3	250.14
254.25539	98		
256.05643	809		
256.9209	246		
258.0965	41		
259.14572	665		
260.42462	307		
261.23996	307		
266.97266	364		
268.12994	198		
269.15613	112		
270.42682	223		
271.10272	1768		
272.11456	149		
273.19382	55		
274.03925	944		
276.98929	1139		

277.7641	306		
278.3692	98		
280.34586	34		
281.30243	85		
283.30646	25		
283.94489	89		
286.33655	190		
287.16193	59		
288.09241	452		
289.05048	499		
291.23413	65		
292.22198	73		
293.17249	52		
295.30255	567		
296.22638	603		
298.03711	451		
299.19769	418		
301.34967	35		
303.90079	771	b_6^{2+}	303.9
304.53839	27		
305.98431	50		
307.05878	99		
309.2959	76		
310.15387	58		
312.32748	733		
313.27631	899		
316.03394	5402	$b_3^+-H_2O$	316.03
317.20123	522		
318.52521	29		
322.39569	101		
323.09332	116		
324.16467	753	$b_7^{2+}-H_2O$	324.16
326.06604	886		
326.96713	183		
328.25842	468		
330.06256	251		
331.21127	407		
332.28436	139		
334.02838	2597	b_3^+	334.03
334.64584	131		
336.27301	33		

337.12933	100		
340.27203	213		
341.19708	33		
342.37866	103		
343.26563	305		
344.17615	122		
345.61719	305		
346.22403	371		
347.48169	56		
348.26239	58		
350.27618	33		
351.28174	189		
352.13336	162		
353.32434	30		
354.26788	72		
355.29407	475		
356.25616	166		
357.01495	120		
358.22528	1185		
359.12973	171		
361.11621	204		
363.30719	52		
364.1839	57		
365.16986	97		
367.05524	752		
369.94122	433		
371.22662	281		
372.18762	70		
373.17645	845		
374.05981	114		
375.12164	546		
376.21436	269		
377.28149	49		
378.3244	178		
379.21863	215		
380.58173	47		
381.19617	226		
381.85876	131		
383.22876	200		
384.23462	50		
385.21088	430		

386.23297	190		
387.18231	64		
388.16382	206		
388.9043	63		
392.1261	31		
393.33368	566		
394.02643	66		
395.31256	1666		
396.19611	341		
397.18243	320		
399.17377	616		
400.44519	156		
401.39874	162		
403.18738	4280	b_4^+-H_2O	403.19
404.12152	404		
405.27777	125		
406.60107	236		
407.43292	260		
408.21045	99		
408.99451	121		
410.04718	67		
412.06165	119		
414.26495	10492	y_3^+	414.26
415.43121	1454		
416.46057	134		
418.16589	376		
419.28778	77		
421.23834	5446	b_4^+	421.24
422.22638	324		
423.59924	458		
424.50208	1003		
425.19775	1705	b_9^{2+}	425.2
426.27399	305		
427.06946	301		
428.25342	114		
429.07361	77		
430.19763	143		
431.7464	269		
432.40338	830		
433.25012	479		
435.19208	24		

437.60291	58		
438.42383	117		
439.18256	30		
441.60333	789		
442.22388	8381		
443.33441	638		
444.04059	345		
445.26154	398		
446.38287	432		
447.14307	164		
450.2818	857		
450.97345	131		
452.26849	151		
454.37238	261		
455.13287	307		
455.98157	52		
457.47528	56		
458.34229	96		
459.71783	865		
460.36395	2717	b_5^+-H_2O	460.36
461.3501	342		
462.27991	203		
464.0033	1051	y_9^{2+}-H_2O, y_9^{2+}-NH_3	464
465.04108	55		
466.41522	84		
468.87201	65		
470.24323	408		
471.08282	217		
472.13586	921	y_9^{2+}	472.14
473.2442	383		
474.3324	149		
475.33771	505		
478.08325	1782	b_5^+	478.08
479.24731	392		
480.41199	188		
481.24493	374		
482.31714	835		
483.37299	254		
484.34479	216		
487.41248	36		
488.20081	252		

490.35046	219		
491.19794	102		
494.2207	70		
496.15625	210		
496.85944	338		
497.46887	75		
498.0943	35		
499.15753	1067		
502.25464	362		
503.32391	88		
505.39185	144		
506.1579	130		
507.4472	116		
508.39105	656		
509.3139	121		
510.46381	131		
511.28259	316		
512.15704	303		
514.0957	1009		
515.34583	12419	y_4^+	515.35
516.34766	1153		
517.25476	437		
518.94543	86		
519.59088	148		
520.22498	441		
521.44891	31		
522.37506	60		
523.30981	130		
525.62012	387		
526.25824	1337		
527.3255	319		
528.73065	442		
529.37042	927		
530.35181	275		
531.33636	355		
532.37415	184		
533.66998	363		
535.37634	437		
536.16931	164		
537.34589	191		
538.26019	382		

539.49933	238		
540.31714	170		
541.41608	646		
542.66663	543		
543.58527	865		
544.27625	1590		
545.34314	602		
546.76044	119		
548.23285	593		
549.37256	26		
551.57953	64		
553.30988	683		
554.27069	947		
555.08575	451		
556.78302	579		
557.42377	100		
558.28424	41		
559.40332	907		
560.25677	184		
561.20123	1677		
562.29596	1275		
563.59088	182		
565.79608	3319	y_{11}^{2+}	565.8
566.51471	640		
568.39728	1863	y_5^+-H_2O	568.4
569.32928	469		
571.20721	2940		
572.21777	2698		
573.06793	133		
574.07965	119		
574.97858	120		
576.38458	4149		
577.75903	256		
579.43671	1684		
580.41766	415		
581.45441	728		
582.6394	486		
583.5329	282		
585.30377	628		
586.3855	13519	y_5^+	586.39
587.47479	1908		

589.22754	12700	b_6^+-H_2O	589.23
590.32458	1922		
591.38361	244		
592.41852	874		
593.32428	632		
594.33289	135		
596.26666	81		
597.57526	93		
598.3244	157		
599.42841	128		
600.58221	2392	y_{12}^{2+}-H_2O, y_{12}^{2+}-NH_3	600.58
601.41888	184		
602.77588	249		
603.53949	1175		
604.2619	89		
606.10529	809		
607.33936	5335	b_6^+	607.34
608.39496	1496		
609.51813	12750	y_{12}^{2+}	609.52
610.43292	981		
611.28052	1378		
612.40051	201		
613.10822	278		
614.87689	4490		
616.32874	383		
617.24335	61		
618.29626	486		
619.07837	253		
619.87231	206		
621.8974	246		
623.08698	64		
624.12952	271		
625.35559	351		
627.24274	1981		
628.25586	5094		
629.19226	1349		
629.95282	278		
631.04828	2471		
632.33429	479		
633.29333	186		
634.73248	183		

635.45074	34		
636.18097	216		
637.00848	135		
638.2146	252		
639.73334	385		
641.08167	994		
642.38373	848		
643.53522	250		
644.40814	123		
645.53082	748		
646.46466	2909	b_7^+-H_2O	646.46
647.39545	1176		
648.56012	188		
650.11554	6750	y_{13}^{2+}-H_2O, y_{13}^{2+}-NH_3	650.12
651.31757	259		
652.36176	134		
653.48871	80		
654.8017	60		
655.78644	343		
656.44568	286		
657.49261	234		
659.00067	38366	y_{13}^{2+}	659
660.25836	1052		
661.10474	1277		
662.0968	52		
663.01733	206		
664.25439	1397	b_7^+	664.25
665.28284	466		
666.31561	28		
667.37384	48		
668.28534	222		
670.25018	363		
672.48358	1277		
673.5119	620		
674.3316	373		
674.97406	56		
675.97595	212		
676.80975	151		
677.94611	261		
678.94202	116		
679.55414	95		

680.93042	36		
682.05621	48		
683.48999	471		
684.34344	387		
685.44818	1421		
686.29333	130		
688.54962	28		
689.47388	220		
691.33807	1030		
692.80798	398		
693.49634	1299	y_{14}^{2+}-H_2O, b_{14}^{2+}-H_2O, y_{14}^{2+}-NH_3	693.5
694.33258	422		
695.49628	312		
696.4809	423		
697.35754	85		
698.31006	136		
699.3858	214		
700.32007	602		
701.41669	7901	y_6^+	701.42
702.54486	4990	y_{14}^{2+}, b_{14}^{2+}	702.54
703.7337	586		
704.61786	201		
705.91132	140		
707.72723	444		
708.36017	210		
709.52313	112		
711.51129	395		
712.51434	394		
713.4599	458		
714.44147	120		
716.21356	297		
717.86389	409		
718.6236	267		
720.5014	317		
721.41754	461		
722.53644	272		
723.46088	182		
726.04486	1598		
727.32336	271		
729.29614	171		
730.25909	769		

731.45709	1449		
732.32666	158		
733.66046	89		
735.89117	459		
736.55499	793		
738.90698	2349		
739.5293	2576		
740.39716	2520	y_7^+-H_2O	740.4
741.44867	772	y_7^+-NH_3	741.45
742.51447	336		
743.71405	341		
744.84344	818		
746.09821	3352		
747.67059	4330		
748.31647	16108		
749.64685	675		
750.62006	400		
752.03937	335		
753.20782	461		
754.30798	783		
754.99823	99		
755.7038	67		
757.47321	7729		
758.37616	62407	y_7^+	758.38
759.43445	11226		
761.30859	658		
762.4162	367		
763.53326	1198		
764.31952	4657		
765.36652	587		
767.07306	40631		
768.08368	912		
769.1485	162		
769.88922	152		
770.51398	34		
771.65265	131		
775.15985	115		
775.97314	58		
776.61859	39		
780.41321	345		
782.60089	177		

783.56329	34		
784.73492	67		
786.45404	420		
787.88153	864		
788.61713	7146		
793.33942	4268	b_8^+	793.34
794.28278	858		
794.961	78		
796.53436	206		
797.58722	182		
798.4679	44		
799.9798	325		
800.65204	87		
801.42334	72		
802.50555	370		
803.33453	121		
804.45258	408		
805.58777	51		
807.54224	48		
808.72906	60		
813.53632	88		
814.3653	617		
815.6153	308		
817.42889	115		
818.50232	144		
819.70111	33		
820.80554	49		
821.5199	25		
822.33002	142		
824.10504	114		
824.71124	38		
828.56488	80		
829.67126	67		
830.92157	169		
832.34302	2984	b_9^+-H_2O	832.34
833.26471	941		
835.74841	59		
837.39642	36		
838.49072	185		
844.6886	50		
846.41669	109		

847.28607	48		
848.2995	51		
850.29089	2295	b_9^+	850.29
851.3941	444		
853.17706	355		
854.38214	649		
855.01489	34		
856.02863	154		
857.37866	61		
858.81451	154		
861.91382	81		
864.53949	59		
869.47461	1967	y_8^+-H_2O	869.47
870.64185	831	y_8^+-NH_3	870.64
871.43646	1028		
872.31793	268		
873.61102	46		
875.87006	121		
879.27252	139		
880.48126	260		
885.56085	147		
887.49042	12857	y_8^+	887.49
888.51343	3371		
889.55524	874		
890.71344	27		
891.41626	42		
895.31891	95		
897.14117	55		
898.03461	264		
898.65454	129		
900.08282	82		
900.70184	57		
901.414	123		
903.11011	171		
904.22308	237		
907.61963	1325		
908.82593	116		
909.57599	125		
910.81464	132		
911.71747	192		
912.84766	30		

915.51984	58		
916.39594	34		
918.60504	221		
919.28638	209		
920.24298	346		
921.65619	169		
922.58563	31		
923.54999	63		
925.50616	2903		
926.54913	2196	y_9^+-H_2O	926.55
927.64142	783	y_9^+-NH_3	927.64
929.30292	824		
930.44727	408		
932.19928	57		
933.48102	304		
934.60431	136		
937.23944	126		
939.63989	140		
942.45807	31		
944.49731	70429	y_9^+	944.5
945.53351	18090		
946.52765	159		
947.51563	1602	b_{10}^+-H_2O	947.52
948.46832	360		
949.65527	42		
950.95581	109		
952.51337	209		
954.26105	569		
955.03778	290		
956.93365	101		
957.64459	82		
958.4389	92		
959.35101	30		
960.87799	280		
965.37115	10360	b_{10}^+	965.37
966.51001	2793		
972.32965	530		
973.55646	240		
976.38068	3756		
978.52216	485		
979.50122	176		

981.50684	162		
982.82538	472		
983.7002	216		
984.44489	151		
985.82147	23		
989.2674	234		
990.50238	396		
996.1272	137		
997.4234	243		
998.08936	51		
1000.44891	1180		
1001.52228	269		
1002.30145	55		
1003.75415	174		
1005.33563	47		
1008.43951	597		
1009.41992	80		
1014.64551	500		
1015.53094	117		
1016.42389	195		
1017.85565	2005		
1018.46271	4143	b_{11}^{+}-H_2O	1018.46
1019.31171	967		
1020.16364	224		
1021.74219	53		
1024.53699	132		
1026.60413	2525		
1031.13062	155		
1031.95532	206		
1032.86816	331		
1033.65186	398		
1034.50525	56		
1035.63074	464		
1036.34277	4059	b_{11}^{+}	1036.34
1037.4696	912		
1038.47437	177		
1040.81689	128		
1047.53613	91		
1048.4624	48		
1053.60962	80		
1055.53149	3573	y_{10}^{+}-H_2O	1055.53

1056.62903	783	y_{10}^{+}-NH_3	1056.63
1057.6377	372		
1059.8468	182		
1060.53955	55		
1064.37341	151		
1065.39014	208		
1068.20557	51		
1069.71362	35		
1071.84473	53		
1073.51514	19353	y_{10}^{+}	1073.52
1074.64136	4645		
1075.72168	33		
1077.5321	56		
1081.42712	106		
1083.22009	556		
1084.36157	187		
1085.44312	222		
1086.67517	57		
1089.27441	202		
1091.23291	164		
1095.02051	241		
1096.03125	506		
1096.82861	23		
1099.27515	59		
1101.47266	2827		
1102.55798	647		
1103.43982	52		
1108.38623	40		
1109.58667	41		
1110.52551	147		
1112.49731	2146	y_{11}^{+}-H_2O	1112.5
1113.60486	1356	y_{11}^{+}-NH_3	1113.6
1114.42249	213		
1119.37573	4486	b_{12}^{+}-H_2O	1119.38
1120.45166	839		
1122.98706	62		
1125.76184	404		
1128.5376	122		
1130.55005	39612	y_{11}^{+}	1130.55
1131.58765	11268		
1135.32056	92		

1137.45435	4426	b_{12}^{+}	1137.45
1138.50854	1392		
1139.24487	116		
1143.38965	409		
1144.47778	113		
1145.09485	75		
1146.74194	255		
1147.78845	215		
1149.39282	38		
1152.15381	63		
1153.26123	142		
1154.7251	42		
1155.33386	99		
1156.02979	87		
1157.50635	867		
1161.54285	2422		
1162.56042	576		
1163.8446	252		
1164.52417	1940		
1165.55652	323		
1167.32227	78		
1168.13196	131		
1169.74353	43		
1170.74194	124		
1171.97241	42		
1176.6344	44		
1181.78198	404		
1182.67432	622		
1183.45679	262		
1184.27478	126		
1192.31909	115		
1193.65112	303		
1195.57837	90		
1197.93152	56		
1199.50293	5204	y_{12}^{+}-H_2O	1199.5
1200.54089	1783	y_{12}^{+}-NH_3	1200.54
1201.47217	139		
1202.71118	58		
1209.50464	40		
1212.13843	25		
1214.45129	40		

1215.52295	63		
1217.53101	55246	y_{12}^{+}	1217.53
1218.5542	16726		
1219.54663	148		
1221.30273	32		
1222.24341	44		
1227.48523	107		
1228.37671	105		
1229.41052	110		
1230.49072	46		
1231.63501	26		
1235.2699	144		
1237.7395	103		
1240.90686	59		
1242.64514	91		
1247.72729	108		
1254.99829	97		
1255.81848	30		
1256.73181	141		
1257.45862	83		
1258.89087	263		
1260.74805	272		
1261.78369	61		
1263.93079	126		
1265.39563	83		
1272.29968	132		
1273.33887	509		
1273.98047	584		
1274.77625	293		
1275.53467	111		
1276.44934	130		
1282.61072	156		
1285.75452	25		
1290.453	27		
1291.40869	538		
1292.56067	3385		
1293.74048	362		
1294.50623	377		
1295.57129	189		
1298.41101	392		
1299.61536	235		

1301.24573	127		
1302.14343	163		
1302.78625	34		
1303.38855	138		
1304.90076	126		
1311.48291	2480		
1312.5636	960		
1313.58057	120		
1316.06042	534		
1316.672	3155	y_{13}^{+}	1316.67
1317.62598	1709		
1319.45398	223		
1322.69885	64		
1325.35071	165		
1326.91052	78		
1329.47168	11065	b_{13}^{+}-H_2O	1329.47
1330.573	3797		
1331.61365	38		
1334.29321	155		
1344.15833	64		
1346.95325	1172		
1347.59045	7485	b_{13}^{+}	1347.59
1348.63062	3330		
1350.89087	34		
1356.41907	78		
1360.30896	132		
1367.56262	83		
1369.42566	219		
1377.85925	70		
1384.87195	37		
1385.78674	252		
1386.61487	1582	b_{14}^{+}-H_2O, y_{14}^{+}-NH_3	1386.61
1387.43274	614		
1388.47119	68		
1396.4967	24		
1403.59167	656		
1404.57837	3375	b_{14}^{+}	1404.58
1405.71033	2997		
1406.73584	212		
1413.68982	455		
1414.55652	203		

1422.30591	153		
1423.31604	453		
1423.98169	185		
1512.995	34		
1543.02051	63		

Supplementary Table 2. Primers list

Number	Purpose	Sequence
1	tRNA promoter region amplification for Gibson. 5’ overlap to pBEST vector. Forward primer.	CCTTTCTGTGGTGAAACCGGATGCTGCAATTCTACTCACGATGAGTGTCCAC
2	tRNA promoter region amplification for Gibson. 5’ overlap for Pyl tRNA synthesis. Reverse primer.	AACCCGGCTGAACGGATTTAGAGTCCATTCGATCTACATGATCAGGTTTCCACTAAGTTGAACATCTTGGG
3	tRNA terminator region amplification for Gibson. 5’ overlap for Pyl tRNA synthesis. Forward primer.	GAATGGACTCTAAATCCGTTCAGCCGGGTTAGATTCCCGGGGTTTCCGCCATTCTCTAAGCATTTTTGGAATAAAAC
4	tRNA terminator region amplification for Gibson. 5’ overlap to pBEST vector. Reverse primer.	GGTCTTCTGCTGTCCCCCACTTGCTGCCGCTCTGTTAAAGAGCTATGTTTCTTCACAAGG
5	pBEST vector amplification. Forward primer.	AGAGCGGCAGCAAGTGGGGGACAGCAGAAG
6	pBEST vector amplification. Reverse primer.	GAATTGCAGCATCCGGTTTC
7	pRaGE Pyl tRNA vector amplification. Forward primer.	TACTCACGATGAGTGTCCAC

8	Yeast origin and selection amplification for yeast assembly. 5' overlap to pRaGE Pyl tRNA vector. Forward primer.	AATCCTCTGACCAGCCAGAAAACGACCTTTCTGTGGTGAAACCGGAT GCTGCAATTCGGATCCGCGAATACCGCTTCCAC
9	Yeast origin and selection amplification. Reverse primer.	GTTTTGCTGGCCGCATCTTC
10	GluRS promoter region amplification for yeast assembly. 5' overlap to yeast selection marker URA3. Forward primer.	CGTTACAGAAAAGCAGGCTGGGAAGCATATTTGAGAAGATGCGGCC AGCAAAACGGATCCGCTTAAACCTTACTGCAAGG
11	GluRS promoter region amplification for yeast assembly. 5' overlap to PylRS. Reverse primer.	CGACTCATCCACAGACCAGTAGCAGAGATCAGAGTGTTTAGTGGTTT TTTATCCATTTGAGTTCCTTAGGGTTAATGTTC
12	GluRS terminator region amplification for yeast assembly. 5' overlap to PylRS. Forward primer.	AAACATCAAACGTGCCGCCCGTTCTGAATCGTATTATAACGGGATTT CTACCAACCTGTAATTCAATTGCATAGATATTC
13	GluRS terminator region amplification for yeast assembly. 5' overlap to tRNA promoter in vector. Reverse primer.	TTCTATCTCTTAAACTTCATAAACCATATCAATCTGTGTGGACACTCA TCGTGAGTATTGCGCCAAAAGTCACAACAAAG
14	PylRS amplification. Forward primer.	ATGGATAAAAAACCACTAAACACTCTG

15	PylRS amplification. Reverse primer	TTACAGGTTGGTAGAAATCC
16	GluRS terminator amplification for yeast assembly. 5’ overlap to pRaGE Pyl TAG vector. Reverse primer.	GGTCTTCTGCTGTCCCCCACTTGCTGCCGCTCTATCTGTTCACTACTTTCGC

Plasmids and genes used in this study

pRaGE Pyl TAG Pr1 deGFP Y35TAG NHis:

GCGGATACATATTTGAATGTATTTAGAAAAATAAACAAATAGGGGTTCCGCGCACATTTCCCCGAAAAGTG
CCACCTGACGTCTAAGAAACCATTATTATCATGACATTAACCTATAAAAATAGGCGTATCACGAGGCCCTT
TCGTCTTCAAGAATTCTGGCGAATCCTCTGACCAGCCAGAAAACGACCTTTCTGTGGTGAAACCGGATGCT
GCAATTCGGATCCGCGAATACCGCTTCCACAAACATTGCTCAAAAGTATCTCTTTGCTATATATCTCTGTGC
TATATCCCTATATAACCTACCCATCCACCTTTCGCTCCTTGAACTTGCATCTAAACTCGACCTCTACATTTTT
TATGTTTATCTCTAGTATTACTCTTTAGACAAAAAAATTGTAGTAAGAACTATTCATAGAGTGAATCGAAAA
CAATACGAAAATGTAAACATTTCCTATACGTAGTATATAGAGACAAAATAGAAGAAACCGTTCATAATTTT
CTGACCAATGAAGAATCATCAACGCTATCACTTTCTGTTCACAAAGTATGCGCAATCCACATCGGTATAGA
ATATAATCGGGGATGCCTTTATCTTGAAAAAATGCACCCGCAGCTTCGCTAGTAATCAGTAAACGCGGGAA
GTGGAGTCAGGCTTTTTTTATGGAAGAGAAAATAGACACCAAAGTAGCCTTCTTCTAACCTTAACGGACCT
ACAGTGCAAAAAGTTATCAAGAGACTGCATTATAGAGCGCACAAAGGAGAAAAAAAGTAATCTAAGATGC
TTTGTTAGAAAAATAGCGCTCTCGGGATGCATTTTTGTAGAACAAAAAAGAAGTATAGATTCTTTGTTGGTA
AAATAGCGCTCTCGCGTTGCATTTCTGTTCTGTAAAAATGCAGCTCAGATTCTTTGTTTGAAAAATTAGCGC
TCTCGTCGCGTTGCATTTTTGTTTTACAAAAATGAAGCACAGATTCTTCGTTGGTAAAATAGCGCTTTCGCG
TTGCATTTCTGTTCTGTAAAAATGCAGCTCAGATTCTTTGTTTGAAAAATTAGCGCTCTCGCGTTGCATTTTT
GTTCTACAAAATGAAGCACAGATGCTTCGTTAACAAAGATATGCTATTGAAGTGCAAGATGGAAACGCAG
AAAATGAACCGGGGATGCGACGTGCAAGATTACCTATGCAATAGATGCAATAGTTTCTCCAGGAACCGAA
ATACATACATTGTCTTCCGTAAAGCGCTAGACTATATATTATTATACAGGTTCAAATATACTATCTGTTTCA
GGGAAAACTCCCAGGTTCGGATGTTCAAAATTCAATGATGGGTAACAAGTACGATCGTAAATCTGTAAAAC
AGTTTGTCGGATATTAGGCTGTATCTCCTCAAAGCGTATTCGAATATCATTGAGAAGCTGCAGCGTCACATC
GGATAATAATGATGGCAGCCATTGTAGAAGTGCCTTTTGCATTTCTAGTCTCTTTCTCGGTCTAGCTAGTTTT
ACTACATCGCGAAGATAGAATCTTAGATCACACTGCCTTTGCTGAGCTGGATCAATAGAGTAACAAAAGAG
TGGTAAGGCCTCGTTAAAGGACAAGGACCTGAGCGGAAGTGTATCGTACAGTAGACGGAGTATCTAGTAT
AGTCTATAGTCCGTGGAATTAATTCTCATCTTTGACAGCTTATCATCGATAAGCTAGCTTTTCAATTCAATTC
ATCATTTTTTTTTTATTCTTTTTTTTGATTTCGGTTTCTTTGAAATTTTTTTGATTCGGTAATCTCCGAACAGA
AGGAAGAACGAAGGAAGGAGCACAGACTTAGATTGGTATATATACGCATATGTAGTGTTGAAGAAACATG
AAATTGCCCAGTATTCTTAACCCAACTGCACAGAACAAAAACCTGCAGGAAACGAAGATAAATCATGTCG
AAAGCTACATATAAGGAACGTGCTGCTACTCATCCTAGTCCTGTTGCTGCCAAGCTATTTAATATCATGCAC
GAAAAGCAAACAAACTTGTGTGCTTCATTGGATGTTCGTACCACCAAGGAATTACTGGAGTTAGTTGAAGC
ATTAGGTCCCAAAATTTGTTTACTAAAAACACATGTGGATATCTTGACTGATTTTTCCATGGAGGGCACAGT
TAAGCCGCTAAAGGCATTATCCGCCAAGTACAATTTTTTACTCTTCGAAGACAGAAAATTTGCTGACATTGG
TAATACAGTCAAATTGCAGTACTCTGCGGGTGTATACAGAATAGCAGAATGGGCAGACATTACGAATGCAC
ACGGTGTGGTGGGCCCAGGTATTGTTAGCGGTTTGAAGCAGGCGGCAGAAGAAGTAACAGAGGAACCTAG
AGGCCTTTTGATGTTAGCAGAATTGTCATGCAAGGGCTCCCTATCTACTGGAGAGTATACTAAGGGTACTGT
TGACATTGCGAAGAGCGACAAAGATTTTGTTATCGGCTTTATTGCTCAAAGAGACATGGGTGGAAGAGATG
AAGGTTACGATTGGCTGATTATGACACCCGGTGTGGGTTTAGATGACAAGGGAGACGCATTGGGTCAACAG
TATAGAACCGTGGATGATGTGGTCTCTACAGGATCTGACATTATTATTGTTGGAAGAGGACTATTTGCAAA
GGGAAGGGATGCTAAGGTAGAGGGTGAACGTTACAGAAAAGCAGGCTGGGAAGCATATTTGAGAAGATGC
GGCCAGCAAAACGGATCCGCTTAAACCTTACTGCAAGGGTAAGGTCAAGGTTTGTTATCAATGAAGTTAAT
TTCGTTGAACCCCGCATCGAGAGGTTATTTGGGTATATGAACACGGAAGGATTGTAGTATTGACTTACGAT
GGTAAAATGTCGCCACTTTTATCCTTGACAATACAACGAGAACCCTCTCGTTGAACATTAACCCTAAGGAA
CTCAAATGGATAAAAAACCACTAAACACTCTGATCTCTGCTACTGGTCTGTGGATGAGTCGTACCGGAACC
ATTCATAAAATCAAACACCACGAGGTTAGCCGTTCGAAAATCTATATTGAGATGGCGTGTGGCGATCATCT
GGTTGTGAACAATAGCCGCTCTTCTCGTACAGCACGTGCACTGCGTCACCACAAATATCGTAAAACCTGTA
AACGTTGCCGTGTGTCCGATGAGGATCTGAACAAATTCCTGACAAAAGCCAATGAGGACCAAACAAGCGT
GAAAGTGAAAGTCGTTAGCGCTCCTACCCGTACTAAAAAAGCAATGCCGAAATCCGTTGCTCGTGCCCCTA
AACCACTGGAAAACACTGAAGCAGCACAGGCACAGCCGTCTGGAAGCAAATTCTCTCCGGCCATTCCTGTT
TCTACCCAGGAGTCCGTTTCTGTTCCAGCAAGTGTGAGCACCAGCATTAGCAGTATTAGCACCGGTGCCAC
CGCTAGCGCCCTGGTTAAAGGCAATACCAATCCGATTACAAGCATGTCTGCCCCGGTTCAAGCATCAGCTC
CAGCACTGACAAAATCCCAAACCGATCGTCTGGAGGTTCTGCTGAATCCGAAAGACGAAATCAGCCTGAAT

TCCGGCAAACCGTTTCGTGAACTGGAGAGCGAACTGCTGTCACGTCGTAAAAAGACCTGCAACAAATCTA
TGCCGAAGAACGTGAGAACTATCTGGGGAAACTGGAACGTGAAATCACCCGCTTTTTCGTGGATCGTGGCT
TTCTGGAGATCAAATCCCCGATTCTGATTCCTCTGGAGTATATCGAGCGTATGGGCATCGACAATGATACCG
AACTGAGCAAACAAATTTTCCGTGTGGATAAAAACTTCTGTCTGCGCCCTATGCTGGCACCAAATCTGTATA
ACTATCTGCGCAAACTGGACCGTGCCCTGCCTGATCCTATCAAAATCTTCGAGATCGGCCCGTGTTATCGTA
AAGAGTCCGACGGTAAAGAACATCTGGAGGAGTTTACCATGCTGAACTTTTGCCAAATGGGTTCAGGTTGT
ACTCGTGAGAACCTGGAAAGCATCATCACCGATTTTCTGAACCACCTGGGCATTGACTTCAAAATTGTGGG
CGACAGCTGTATGGTGTATGGCGACACCCTGGATGTCATGCACGGCGACCTGGAACTGTCTAGTGCCGTTG
TTGGACCAATTCCGCTGGACCGTGAGTGGGGTATCGACAAACCGTGGATCGGAGCAGGATTCGGTCTGGAA
CGCCTGCTGAAAGTGAAACACGACTTCAAAAACATCAAACGTGCCGCCCGTTCTGAATCGTATTATAACGG
GATTTCTACCAACCTGTAATTCAATTGCATAGATATTCAAAAACCGCGGTAATTTCCGCGGTTTTTTATGTC
TAAAGAATGTAATAAAAGCGAAAGTAGTGAACAGATTTGACTGATGTACATGCTATGTCGGCGTGTTGATG
TTTGTTTTTGATAAATTTTTGTAATTAAAGATCGTGGTTTTAGTGCTTATTCAATGGCTGATTTTCAAACT
ATCCCTCTGATTAATATCTACTATCTATAAAGTTCCCCAAGTGGCATCTCAATAGTGAGACAAATATCTGCA
TTAGTTACTTTGTTGTGACTTTTGGCGCAATACTCACGATGAGTGTCCACACAGATTGATATGGTTTATGAA
GTTTAAGAGATAGAACATCCCCCAAGATGTTCAACTTAGTGGAAACCTGATCATGTAGATCGAATGGACTC
TAAATCCGTTCAGCCGGGTTAGATTCCCGGGGTTTCCGCCATTCTCTAAGCATTTTGGAATAAAACGTCCA
AACCAGAGCAAGCAATTGACACTGGTTGGCATTTTTGACTCTGAAAGTCTTTAGAAAATGTACTTCCTCGTG
AAGAAACATAGCTCTTTAACAGAGCGGCAGCAAGTGGGGGACAGCAGAAGACCTGACCGCCGCAGAGTGG
ATGTTTGACATGGTGAAGACTATCGCACCATCAGCCAGAAAACCGAATTTTGCTGGGTGGGCTAACGATAT
CCGCCTGATGCGTGAACGTGACGGACGTAACCACCGCGACATGTGTGTGCTGTTCCGCTGGGCATGCTGAG
CTAACACCGTGCGTGTAGACAATTTTACCTCTGGCGGTGATAATGGTTGCAGCTAGCAATAATTTTGTTTAA
CTTTAAGAAGGAGATATACCATGGGCAGCAGCCATCATCATCATCATCACAGCTCTAGAGAGCTTTTCACT
GGCGTTGTTCCCATCCTGGTCGAGCTGGACGGCGACGTAAACGGCCACAAGTTCAGCGTGTCCGGCGAGGG
CGAGGGCGATGCCACCTAGGGCAAGCTGACCCTGAAGTTCATCTGCACCACCGGCAAGCTGCCCGTGCCCT
GGCCCACCCTCGTGACCACCCTGACCTACGGCGTGCAGTGCTTCAGCCGCTACCCCGACCACATGAAGCAG
CACGACTTCTTCAAGTCCGCCATGCCCGAAGGCTACGTCCAGGAGCGCACCATCTTCTTCAAGGACGACGG
CAACTACAAGACCCGCGCCGAGGTGAAGTTCGAGGGCGACACCCTGGTGAACCGCATCGAGCTGAAGGGC
ATCGACTTCAAGGAGGACGGCAACATCCTGGGGCACAAGCTGGAGTACAACTACAACAGCCACAACGTCT
ATATCATGGCCGACAAGCAGAAGAACGGCATCAAGGTGAACTTCAAGATCCGCCACAACATCGAGGACGG
CAGCGTGCAGCTCGCCGACCACTACCAGCAGAACACCCCCATCGGCGACGGCCCCGTGCTGCTGCCCGACA
ACCACTACCTGAGCACCCAGTCCGCCCTGAGCAAAGACCCCAACGAGAAGCGCGATCACATGGTCCTGCTG
GAGTTCGTGACCGCCGCCGGGATCTGACTCGAGCAAAGCCCGCCGAAAGGCGGGCTTTTCTGTGTCGACCG
ATGCCCTTGAGAGCCTTCAACCCAGTCAGCTCCTTCCGGTGGGCGCGGGGCATGACTATCGTCGCCGCACTT
ATGACTGTCTTCTTTATCATGCAACTCGTAGGACAGGTGCCGGCAGCGCTCTTCCGCTTCCTCGCTCACTGA
CTCGCTGCGCTCGGTCGTTCGGCTGCGGCGAGCGGTATCAGCTCACTCAAAGGCGGTAATACGGTTATCCA
CAGAATCAGGGGATAACGCAGGAAAGAACATGTGAGCAAAAGGCCAGCAAAAGGCCAGGAACCGTAAAA
AGGCCGCGTTGCTGGCGTTTTTCCATAGGCTCCGCCCCCCTGACGAGCATCACAAAAATCGACGCTCAAGT
CAGAGGTGGCGAAACCCGACAGGACTATAAAGATACCAGGCGTTTCCCCCTGGAAGCTCCCTCGTGCGCTC
TCCTGTTCCGACCCTGCCGCTTACCGGATACCTGTCCGCCTTTCTCCCTTCGGGAAGCGTGGCGCTTTCTCAA
TGCTCACGCTGTAGGTATCTCAGTTCGGTGTAGGTCGTTCGCTCCAAGCTGGGCTGTGTGCACGAACCCCCC
GTTCAGCCCGACCGCTGCGCCTTATCCGGTAACTATCGTCTTGAGTCCAACCCGGTAAGACACGACTTATCG
CCACTGGCAGCAGCCACTGGTAACAGGATTAGCAGAGCGAGGTATGTAGGCGGTGCTACAGAGTTCTTGA
AGTGGTGGCCTAACTACGGCTACACTAGAAGGACAGTATTTGGTATCTGCGCTCTGCTGAAGCCAGTTACC
TTCGGAAAAAGAGTTGGTAGCTCTTGATCCGGCAAACAAACCACCGCTGGTAGCGGTGGTTTTTTTGTTTGC
AAGCAGCAGATTACGCGCAGAAAAAAAGGATCTCAAGAAGATCCTTTGATCTTTTCTACGGGGTCTGACGC
TCAGTGGAACGAAAACTCACGTTAAGGGATTTTGGTCATGAGATTATCAAAAAGGATCTTCACCTAGATCC
TTTTAAATTAAAAATGAAGTTTTAAATCAATCTAAAGTATATATGAGTAAACTTGGTCTGACAGTTACCAAT
GCTTAATCAGTGAGGCACCTATCTCAGCGATCTGTCTATTTCGTTCATCCATAGTTGCCTGACTCCCCGTCGT
GTAGATAACTACGATACGGGAGGGCTTACCATCTGGCCCCAGTGCTGCAATGATACCGCGAGACCCACGCT
CACCGGCTCCAGATTTATCAGCAATAAACCAGCCAGCCGGAAGGGCCGAGCGCAGAAGTGGTCCTGCAAC
TTTATCCGCCTCCATCCAGTCTATTAATTGTTGCCGGGAAGCTAGAGTAAGTAGTTCGCCAGTTAATAGTTT
GCGCAACGTTGTTGCCATTGCTACAGGCATCGTGGTGTCACGCTCGTCGTTTGGTATGGCTTCATTCAGCTC
CGGTTCCCAACGATCAAGGCGAGTTACATGATCCCCCATGTTGTGCAAAAAAGCGGTTAGCTCCTTCGGTC
CTCCGATCGTTGTCAGAAGTAAGTTGGCCGCAGTGTTATCACTCATGGTTATGGCAGCACTGCATAATTCTC
TTACTGTCATGCCATCCGTAAGATGCTTTTCTGTGACTGGTGAGTACTCAACCAAGTCATTCTGAGAATAGT

GTATGCGGCGACCGAGTTGCTCTTGCCCGGCGTCAATACGGGATAATACCGCGCCACATAGCAGAACTTTA
AAAGTGCTCATCATTGGAAAACGTTCTTCGGGGCGAAAACTCTCAAGGATCTTACCGCTGTTGAGATCCAG
TTCGATGTAACCCACTCGTGCACCCAACTGATCTTCAGCATCTTTTACTTTCACCAGCGTTTCTGGGTGAGC
AAAAACAGGAAGGCAAAATGCCGCAAAAAAGGGAATAAGGGCGACACGGAAATGTTGAATACTCATACT
CTTCCTTTTTCAATATTATTGAAGCATTTATCAGGGTTATTGTCTCATGA

V. natriegens native GluRS upstream:

GCTTAAACCTTACTGCAAGGGTAAGGTCAAGGTTTGTTATCAATGAAGTTAATTTCGTTGAACCCCGCATCG
AGAGGTTATTTGGGTATATGAACACGGAAGGATTGTAGTATTGACTTACGATGGTAAAATGTCGCCACTTTT
ATCCTTGACAATACAACGAGAACCCTCTCGTTGAACATTAACCCTAAGGAACTCAA

V. natriegens native GluRS downstream:

TTCAATTGCATAGATATTCAAAAACCGCGGTAATTTCCGCGGTTTTTTTATGTCTAAAGAATGTAATAAAAG
CGAAAGTAGTGAACAGATTTGACTGATGTACATGCTATGTCGGCGTGTTGATGTTTGTTTTTTGATAAATTT
TTGTAATTAAAGATCGTGGTTTTTAGTGCTTATTCAATGGCTGATTTTCAAACTATCCCTCTGATTAATATCT
ACTATCTATAAAGTTCCCCAAGTGGCATCTCAATAGTGAGACAAATATCTGCATTAGTTACTTTGTTGTGAC
TTTTGGCGCAA

*Mm*PylRS gene:

ATGGATAAAAAACCACTAAACACTCTGATCTCTGCTACTGGTCTGTGGATGAGTCGTACCGGAACCATTCA
TAAAATCAAACACCACGAGGTTAGCCGTTCGAAAATCTATATTGAGATGGCGTGTGGCGATCATCTGGTTG
TGAACAATAGCCGCTCTTCTCGTACAGCACGTGCACTGCGTCACCACAAATATCGTAAAACCTGTAAACGT
TGCCGTGTGTCCGATGAGGATCTGAACAAATTCCTGACAAAAGCCAATGAGGACCAAACAAGCGTGAAAG
TGAAAGTCGTTAGCGCTCCTACCCGTACTAAAAAAGCAATGCCGAAATCCGTTGCTCGTGCCCCTAAACCA
CTGGAAAACACTGAAGCAGCACAGGCACAGCCGTCTGGAAGCAAATTCTCTCCGGCCATTCCTGTTTCTAC
CCAGGAGTCCGTTTCTGTTCCAGCAAGTGTGAGCACCAGCATTAGCAGTATTAGCACCGGTGCCACCGCTA
GCGCCCTGGTTAAAGGCAATACCAATCCGATTACAAGCATGTCTGCCCCGGTTCAAGCATCAGCTCCAGCA
CTGACAAAATCCCAAACCGATCGTCTGGAGGTTCTGCTGAATCCGAAAGACGAAATCAGCCTGAATTCCGG
CAAACCGTTTCGTGAACTGGAGAGCGAACTGCTGTCACGTCGTAAAAAAGACCTGCAACAAATCTATGCCG
AAGAACGTGAGAACTATCTGGGGAAACTGGAACGTGAAATCACCCGCTTTTTCGTGGATCGTGGCTTTCTG
GAGATCAAATCCCCGATTCTGATTCCTCTGGAGTATATCGAGCGTATGGGCATCGACAATGATACCGAACT
GAGCAAACAAATTTTCCGTGTGGATAAAAACTTCTGTCTGCGCCCTATGCTGGCACCAAATCTGTATAACTA
TCTGCGCAAACTGGACCGTGCCCTGCCTGATCCTATCAAAATCTTCGAGATCGGCCCGTGTTATCGTAAAGA
GTCCGACGGTAAAGAACATCTGGAGGAGTTTACCATGCTGAACTTTTGCCAAATGGGTTCAGGTTGTACTC
GTGAGAACCTGGAAAGCATCATCACCGATTTCTGAACCACCTGGGCATTGACTTCAAAATTGTGGGCGAC
AGCTGTATGGTGTATGGCGACACCCTGGATGTCATGCACGGCGACCTGGAACTGTCTAGTGCCGTTGTTGG
ACCAATTCCGCTGGACCGTGAGTGGGGTATCGACAAACCGTGGATCGGAGCAGGATTCGGTCTGGAACGCC
TGCTGAAAGTGAAACACGACTTCAAAAACATCAAACGTGCCGCCCGTTCTGAATCGTATTATAACGGGATT
TCTACCAACCTGTAA

V. natriegens native tRNA upstream:

TACTCACGATGAGTGTCCACACAGATTGATATGGTTTATGAAGTTTAAGAGATAGAACATCCCCCAAGATG
TTCAACTTAGT

V. natriegens native tRNA downstream:

TTCTCTAAGCATTTTTGGAATAAAACGTCCAAACCAGAGCAAGCAATTGATACTGGTTGGCATTTTTGACTC
TGAAAGTCTTTAGAAAATGTACTTCCTTGTGAAGAAACATAGCTCTTTAAC

*Mm*Pyl-tRNA:

GGAAACCTGATCATGTAGATCGAATGGACTCTAAATCCGTTCAGCCGGGTTAGATTCCCGGGGTTTCCGCC
A

deGFP Y35TAG NHis gene:

ATGGGCAGCAGCCATCATCATCATCATCACAGCTCTAGAGAGCTTTTCACTGGCGTTGTTCCCATCCTGGTC
GAGCTGGACGGCGACGTAAACGGCCACAAGTTCAGCGTGTCCGGCGAGGGCGAGGGCGATGCCACCTAGG
GCAAGCTGACCCTGAAGTTCATCTGCACCACCGGCAAGCTGCCCGTGCCCTGGCCCACCCTCGTGACCACC
CTGACCTACGGCGTGCAGTGCTTCAGCCGCTACCCCGACCACATGAAGCAGCACGACTTCTTCAAGTCCGC
CATGCCCGAAGGCTACGTCCAGGAGCGCACCATCTTCTTCAAGGACGACGGCAACTACAAGACCCGCGCCG
AGGTGAAGTTCGAGGGCGACACCCTGGTGAACCGCATCGAGCTGAAGGGCATCGACTTCAAGGAGGACGG
CAACATCCTGGGGCACAAGCTGGAGTACAACTACAACAGCCACAACGTCTATATCATGGCCGACAAGCAG
AAGAACGGCATCAAGGTGAACTTCAAGATCCGCCACAACATCGAGGACGGCAGCGTGCAGCTCGCCGACC
ACTACCAGCAGAACACCCCCATCGGCGACGGCCCCGTGCTGCTGCCCGACAACCACTACCTGAGCACCCAG
TCCGCCCTGAGCAAAGACCCCAACGAGAAGCGCGATCACATGGTCCTGCTGGAGTTCGTGACCGCCGCCGG
GATCTGA

*Linking amino acids + His-tag
